# Added Value of Genomic Surveillance of Virulence Factors in Shiga Toxin-Producing *Escherichia coli* in New South Wales, Australia

**DOI:** 10.3389/fmicb.2021.713724

**Published:** 2021-12-23

**Authors:** Eby M. Sim, Ryan Kim, Mailie Gall, Alicia Arnott, Peter Howard, Mary Valcanis, Benjamin P. Howden, Vitali Sintchenko

**Affiliations:** ^1^Enteric Reference Laboratory and Microbial Genomics Laboratory, Centre for Infectious Diseases and Microbiology Laboratory Services, NSW Health Pathology, Institute of Clinical Pathology and Medical Research, Westmead, NSW, Australia; ^2^Microbiological Diagnostic Unit Public Health Laboratory, Department of Microbiology and Immunology, Peter Doherty Institute for Infection and Immunity, The University of Melbourne, Melbourne, VIC, Australia; ^3^Marie Bashir Institute for Infectious Diseases and Biosecurity, The University of Sydney, Sydney, NSW, Australia; ^4^Centre for Infectious Diseases and Microbiology-Public Health, Westmead Hospital, Westmead, NSW, Australia

**Keywords:** STEC, Top-7 serotypes, *stx* subtyping, virulence analysis, cgMLST

## Abstract

The disease caused by Shiga toxin-producing *Escherichia coli* (STEC) remains a significant public health challenge globally, but the incidence of human STEC infections in Australia remains relatively low. This study examined the virulence characteristics and diversity of STEC isolates in the state of New South Wales between December 2017 and May 2020. Utilisation of both whole and core genome multi-locus sequence typing (MLST) allowed for the inference of genomic diversity and detection of isolates that were likely to be epidemiologically linked. The most common STEC serotype and *stx* subtype detected in this study were O157:H7 and *stx*_1a_, respectively. A genomic scan of other virulence factors present in STEC suggested interplay between iron uptake system and virulence factors that mediate either iron release or countermeasures against host defence that could result in a reduction of *stx*_1a_ expression. This reduced expression of the dominant *stx* genotype could contribute to the reduced incidence of STEC-related illness in Australia. Genomic surveillance of STEC becomes an important part of public health response and ongoing interrogation of virulence factors in STEC offers additional insights for the public health risk assessment.

## Introduction

*Escherichia coli* is a bacterial commensal in humans, but some strains have evolved to be pathogens by the acquisition of virulence factors. Shiga toxin-producing *Escherichia coli* (STEC) is a group of predominantly food-borne pathogens that can cause acute gastroenteritis and sometimes haemolytic uraemic syndrome (HUS), neurological complications, and even death (Tarr et al., 2005). This progression to HUS has been linked to the production of Shiga toxin (Stx) encoded by the *stx* operon consisting of two genes encoding the enzymatically active subunit A and a receptor binding pentameric subunit B of the A_1_B_5_ holotoxin, respectively (Melton-Celsa, 2014). STEC can produce two structurally similar but immunologically distinct toxins, namely, Stx1 and Stx2, which can be further subtyped into s*tx*_1a_, s*tx*_1c_, s*tx*_1d_, and s*tx*_2a–2g_ (Melton-Celsa, 2014). This ability to produce Stx is conferred by temperate bacteriophages (*stx* phages) (Allison, 2007). Not all subtypes are equally pathogenic and infections with STEC harbouring either *stx*_2a_, *stx*_2c_, or *stx*_2d_ appeared to be more likely to progress to HUS (Friedrich et al., 2002; Orth et al., 2007). Each *stx* (pro)phage harbours one *stx* operon and the detection of multiple *stx* operons, within an *E. coli* host, is an indication of multiple integrated *stx* prophages (Hayashi et al., 2001; Perna et al., 2001; Allison et al., 2003; Fogg et al., 2007; Forde et al., 2019). The *stx* operon is located in a conserved location within the *stx* phage, linking Stx production with prophage induction (Neely and Friedman, 1998). This switch to the lytic life cycle *via* prophage induction releases infectious phage virions, which can disseminate the *stx* genes to other *E. coli* cells at the expense of its current host (Allison, 2007).

This horizontal dissemination of *stx* has allowed for the emergence of hybrid *E. coli* pathotypes. The most prolific hybrid was the Shiga toxin-producing enteroaggregative *E. coli* (EAEC) responsible for the large community outbreak in Germany associated with contaminated sprouts (Rohde et al., 2011). Apart from producing Stx, some STEC also harboured the locus of enterocyte effacement (LEE), a pathogenicity island also found in both typical and atypical enteropathogenic *E. coli* (EPEC) (Schmidt, 2010). This pathogenicity island encodes a type 3 secretion system (T3SS) responsible for both intimate adhesion mediated by intimin (encoded by the *eae* gene) and its translocated receptor Tir, as well as the effacement of microvilli (Deng et al., 2004; Schmidt, 2010). Not all effector proteins secreted by this T3SS are encoded within the LEE, and these non-LEE effectors can be found either on genomic island or associated with prophages (Deng et al., 2004; Creuzburg et al., 2005; Tobe et al., 2006; Ogura et al., 2009). Some STEC also harboured a plasmid that encodes multiple virulence genes of which the gene *ehxA* (sometimes also referred to as *hlyA*), part of the plasmid borne haemolysin operon, has been traditionally used as a diagnostic marker for STEC in culture (Paton and Paton, 1998; Lim et al., 2010). While each individual virulence gene in this plasmid contribute to virulence in their own right, there has yet to be a direct association of between plasmid carriage and severity of disease (Lim et al., 2010).

Horizontal dissemination of *stx* also enabled virulence among multiple serotypes with STEC serotype O157:H7 being the most commonly recognised as causing human disease (Rangel et al., 2005; Vally et al., 2012; Gould et al., 2013). However, other non-O157 serotypes have also been linked to severe disease, namely, O26, O45, O103, O111, O121, and O145 (Gould et al., 2013; Luna-Gierke et al., 2014). Collectively, these seven serotypes are known as the “Top-7” STEC serotypes, a term used to describe all STEC deemed adulterants by the U.S. Department of Agriculture, Food Safety and Inspection Services (USDA-FSIS, 2011). In addition to serotyping, other molecular techniques, like the lineage specific polymorphism assay (LSPA-6) and the Manning SNP typing scheme, added further resolution but they tended to be biassed to STEC O157:H7 (Yang et al., 2004; Manning et al., 2008). Whole genome sequencing (WGS) has been recently adopted for public health surveillance of STEC and has allowed *in silico* inference of serotypes and detection of STEC outbreaks as well retrospective epidemiological studies (Joensen et al., 2014; Dallman et al., 2015; Ingle et al., 2019; Jenkins et al., 2019).

The incidence of STEC-associated illness in Australia was historically reported as low (Vally et al., 2012), but with increased surveillance using molecular techniques, incidence was determined to be 2.07 per 100,000 population (Supplementary Table 1). Barring differences in detection methodology, the incidence in Australia was comparable to the United Kingdom and Canada while significantly lower than in New Zealand (Supplementary Table 1). The most common serotype isolated in Australia was STEC O157:H- (Vally et al., 2012; Ingle et al., 2019) with the non-motile phenotype associated with a single base insertion in *flgF* gene (Pintara et al., 2018). However, these non-motile O157 did encode an intact *fliC*, the target for *in silico* H-antigen typing, and as such would be genomically identified as O157:H7 when typed *in silico* (Pintara et al., 2018). Australian STEC O157 isolates typically harbour *stx*_1a_ and *stx*_2c_ either alone or in combination (Fegan and Desmarchelier, 2002; Mellor et al., 2012, 2015) and have been reported to be genetically distinct from international collections of isolates (Leotta et al., 2008; Mellor et al., 2012, 2013, 2015; Ingle et al., 2019; Pintara et al., 2020). It remains unknown if the other non-O157 STEC were also geographically and genetically distinct. In this study, we examined genomic diversity and pathogenic potential of STEC isolates recovered from human cases of gastroenteritis and HUS between December 2017 and May 2020 in New South Wales (NSW), Australia using WGS and downstream analysis. While useful in epidemiology, serotyping is not a reliable indicator for the disease states caused by STEC with the best predictors being *stx* subtypes and the presence or absence of *eae* (WHO and FAO, 2018). To that end, we developed a virulence barcode to be used with WGS data, which not only tracks the subtypes of prominent STEC virulence factors *eae* and *stx*, but also allows for the inference of multiple, isogenic *stx* copies derived from short read sequencing data. We also aimed to adapt existing *in silico* tools currently utilised in pathogen genomics, along with an existing wgMLST schema for routine prospective public health laboratory surveillance of STEC.

## Materials and Methods

### Shiga Toxin-Producing *Escherichia coli* Isolation, DNA Extraction, and Whole Genome Sequencing

Shiga toxin-producing *Escherichia coli* isolates were cultured from unenriched faecal samples initially subjected to polymerase chain reaction (PCR) testing on the BD-MAX™ platform using the Enteric Bacterial Panel targetting the *stx* genes (Becton Dickinson). Isolation of STEC colonies was attempted for samples that were PCR positive for *stx*_1_ and/or *stx*_2_, by picking up to three separate colonies and testing these subcultured colonies for the presence of *stx* with subsequent somatic O antigen serotyping at the NSW Enteric Reference Laboratory, the Institute of Clinical Pathology and Medical Research (ICPMR)-NSW Health Pathology, NSW, Australia. STEC isolates collected between December 2017 and December 2019 were also forwarded to the Microbiological Diagnostic Unit Public Health Laboratory (MDU PHL), Victoria, Australia for sequencing as previously described (Ingle et al., 2019). Isolates from January 2020 were sequenced at ICPMR-NSW Health Pathology. DNA was extracted from isolates sequenced at ICPMR using QIAGEN DNeasy Blood & Tissue Kit. Sequencing libraries were prepared using Nextera XT and sequenced on the NextSeq500 platform (Illumina) with 150-bp paired-end reads.

### Shiga Toxin-Producing *Escherichia coli* Genomic Assembly and Virulence Finding

Sequencing reads obtained from both ICPMR and MDU PHL were subsequently trimmed using Trimmomatic version 0.36 (Bolger et al., 2014) and assembled using SKESA version 2.3 (Souvorov et al., 2018) on default settings. When required, both Artemis version 18.1.0 (Carver et al., 2012) and Artemis Comparison Tool (ACT) version 18.1.0 (Carver et al., 2005) were used for visualising draft assemblies and pairwise BLASTN comparison, respectively. ABRicate version 1.0.1^1^ was employed to infer presence (minid 90; mincov 90) of antimicrobial resistance genes against the Resfinder database (Zankari et al., 2012), *E. coli* virulence factors against the ecoli_VF database^2^, and *stx* and *eae* subtypes using a custom database derived from reference sequences in the published literature (Blanco et al., 2005; Scheutz et al., 2012). To aid in data collation post ABRicate screening, *E. coli* virulence genes were clustered using CD-HIT version 4.8.1 (Fu et al., 2012) to collapse (-c 0.9; -g 1) virulence factors into a single group when multiple homologues exist. Assignation of the virulence factors belonging to the various secretomes was performed as previously reviewed (Monteiro et al., 2016). Type 6 Secretion Systems identified were further subtyped based on the SecReT6 Database (Li et al., 2015). Genomic locations of non-LEE effectors were assigned employing locations reported in reference genomes of STEC O157 (Hayashi et al., 2001; Tobe et al., 2006) and non-O157 (Ogura et al., 2009) isolates.

Mapping of fastq reads in this study was performed using BWA version 0.7.17-r1188 with the MEM algorithm (Li, 2013). Normalisation of *stx*, to detect potential harbouring of multiple isogenic *stx* genes (multiple copies of identical *stx* genes), was achieved by mapping sequencing reads onto a pseudomolecule consisting of the aforementioned *stx* subtype database and the nucleotide sequence of *recA* from STEC O157 strain Sakai (NCBI GenBank Accession: BA000007.3) to account for possible interference from the library preparation process. The mean read depth of identified *stx* subtype was normalised against the mean read depth of the housekeeping gene *recA*. All normalised values <2 were reported as unlikely to possess additional isogenic *stx* subtypes. For all values ≥2, the mean read depth of *stx* was further normalised against the “+1 standard deviation” of the mean read depth over *recA* gene sequence. Samples with a normalised value of ≥2 for both were reported as inferred plausible for isogenic *stx* subtypes possession while samples with a value <2 following additional normalisation was reported as inferred possible for multiple isogenic *stx* subtype possession. Published genomes and/or sequencing reads of STEC O157:H7 strain Sakai (Hayashi et al., 2001), STEC O157:H7 strain TW14359 (Kulasekara et al., 2009), STEC O111:H- strain 95NR1 (McAllister et al., 2016; Forde et al., 2019), STEC O104:H4 strain 2011C-3493 (Ahmed et al., 2012), EPEC O127:H6 strain E2348/69 (Iguchi et al., 2009), and K-12 strain MG1655 (Blattner et al., 1997) were used as either controls or reference genomes for mapping.

### Serotyping, Multi-Locus Sequence Typing Typing, and Core Single-Nucleotide Polymorphisms Analysis

Multi-locus sequence typing (MLST) was determined using mlst version 2.19^3^, a software that utilised resources from PubMLST (Jolley and Maiden, 2010) against the Achtman scheme (Wirth et al., 2006). *In silico* O:H serotyping was predicted using ABRicate (minid 95; mincov 30) against the EcOH database (Ingle et al., 2016). Whole genome MLST (wgMLST; 2,513 loci) and core genome MLST (cgMLST; 2,248 loci) were determined using the *E. coli* schema from Ridom^4^ and chewBBACA version 2.5.5 (Silva et al., 2018). In this study, cgMLST was defined as wgMLST loci present amongst all samples within the dataset. Results of both wgMLST and cgMLST were visualised as a minimum spanning tree generated with GrapeTree using the MSTree V2 algorithm (Zhou et al., 2018). Analysis of single-nucleotide polymorphisms (SNP) in STEC genomes was performed using Snippy version 4.6.0^5^ with different reference genomes, chosen according to their serotypes. SNP distance matrices were generated using snp-dists version 0.6^6^.

### Additional *in silico* Typing for Shiga Toxin-Producing *Escherichia coli* O157:H7

*In silico* LSPA-6 PCR of STEC O157 was performed using previously described oligonucleotides (Yang et al., 2004) using isPcr^7^. STEC O157:H7 clades according to the Manning scheme (Manning et al., 2008) were visually determined in the draft assemblies using a set of previously described discriminatory positions (Yokoyama et al., 2012). An older version of STEC O157 strain Sakai genome (NCBI GenBank Accession: BA000007.2) was used as a reference for clade typing instead of the newer version (Accession: BA00007.3) as discrepancies in locus tags were previously documented (Ingle et al., 2019) when the genome of STEC O157 strain Sakai was resequenced. Insertion of cytosine within *flgF* (Pintara et al., 2018), a predicted marker for the non-motile phenotype in Australian STEC O157, was visually interrogated from the draft assembly. Presence of the STEC O157 long polar fimbriae was screened using ABRicate (minid 90; mincov 90) on a custom database consisting of the genes that encoded both the LpfA and the LpfD2 pilus of STEC O157:H7 strain Sakai (Hayashi et al., 2001).

### Development and Deployment of a Shiga Toxin-Producing *Escherichia coli* Virulence Barcode

To provide a quick snapshot of pertinent virulence factors, all STEC isolates were assigned a STEC virulence barcode made up of 12 digits in the format of “XX-XX-XX-XX-XX-XX” reflecting their STEC virulence makeup. Each set of two digits represented a particular virulence factor. The first set of two digits represented the presence (to the subtype level) or absence of the *eae* gene. The next set of two digits represented inference of possible multiple, isogenic *stx* genes not assembled *via* short read sequencing. The last four sets of 2-mers each reflected the presence (to the subtype level) or absence of *stx*. This representation allowed up to four different *stx* operons to be captured, which is currently the maximum number observed both *in vitro* (Fogg et al., 2007) and in isolates (McAllister et al., 2016; Forde et al., 2019). The codex for decoding the virulence barcode is listed in Table 1.

**TABLE 1:** Codex for decoding the 12-mer STEC virulence barcode and detection methodology.

Code	Value	Detection means
***eae* subtyping**		
00	Absent	Presence determined by mapping unto pseudomolecule consisting of *eae* (STEC O157-γ1 subtype), subtyping performed using draft assembly
XX	Untypeable	
01	α1	
02	α2	
03	β1	
04	ξR/β2B	
05	δ/κ/β2O	
06	γ1	
07	γ2/θ	
08	ε1	
09	νR/ε2	
10	ζ	
11	η	
12	ι1	
13	μR/ι2	
14	λ	
15	μB	
16	νB	
17	ξB	
**Inference of additional isogenic toxin**		
00	Unlikely to possess extra copies	Mapping unto pseudomolecule consisting of *stx* subtypes and the *recA* gene of STEC O157 strain Sakai
T1	Inferred possible additional *stx*_1_ subtype	
01	Inferred plausible additional *stx*_1_ subtype	
T2	Inferred possible additional *stx*_2_ subtype	
02	Inferred plausible additional *stx*_1_ subtype	
CG	Genome resolved	
***stx* subtyping**		
00	Absent	Assembly and mapping against a reference consisting of *stx* subtypes
1A	*stx*_1a_	
1C	*stx*_1c_	
1D	*stx*_1d_	
2A	*stx*_2a_	
2B	*stx*_2b_	
2C	*stx*_2c_	
2D	*stx*_2d_	
2E	*stx*_2e_	
2F	*stx*_2f_	
2G	*stx*_2g_	

## Results

### Representativeness of New South Wales Shiga Toxin-Producing *Escherichia coli* Genomic Surveillance

From December 2017 to May 2020, there were 1,570 cases of STEC notifications nationally and 186 of those notifications were from NSW. Within this time period, NSW Enteric Reference laboratory investigated 67 putative STEC (36% of all notifications); 62 of them were subsequently confirmed as STEC while five isolates did not possess the *stx* operon. Majority of the STEC isolates, with the exception of two, had accompanying metadata, which included clinical manifestations. Taken together, our genomic dataset covered 33.3% of all STEC cases notified to NSW Health between December 2017 and May 2020.

### Assembly and *in silico* Screening

**FIGURE 1:**
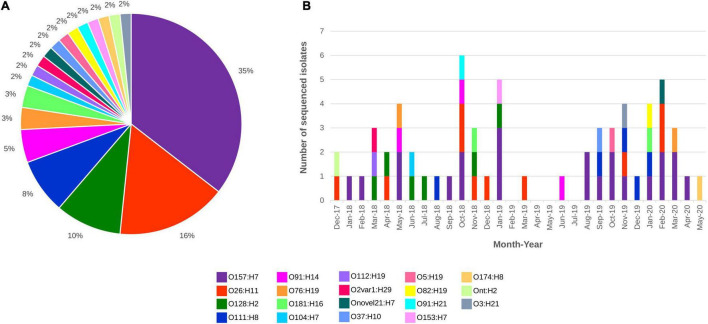
STEC sequenced from December 2017 to May 2020 distributed by **(A)** Serotype and **(B)** Serotype and month of isolation. All serotypes are colour coded as listed in the figure key.

All 62 draft genomes have sizes ranging between 4.75 Mb and 5.58 Mb, within the bounds of an *E. coli* genome (Blattner et al., 1997; Hayashi et al., 2001), with the number of contigs (>300 bp) ranging from 70 to 363 (Supplementary Table 2). Nineteen different serotypes were detected *in silico*, which included three “Top-7 STEC” serotypes, namely, O157:H7, O26:H11, and O111:H8 (Figure 1). Isolates of O157:H7 and O26:H11 were distributed across the study period while some isolates belonging to both O128:H2 and O111:H8 were recovered in close succession to each other. Screening for genes that were involved in antimicrobial resistance (Supplementary Table 3) detected the carriage of *mdfA*, a gene that encodes an efflux pump that when overexpressed, confer resistance to a broad range of antibiotics (Edgar and Bibi, 1997), in all STEC genomes. Two O157:H7 isolates also screened positive for additional AMR markers. The first isolate contained *bla*_TEM–1B_ while the second isolate screened positive for *bla*_TEM–1B_, *strA, strB*, *sul2*, and *tetA*. Of the 62 assemblies, 10 had *stx* genes that were not contiguously assembled, despite mapping showing the presence of the associated *stx* operon. In total, six *stx* subtypes were documented, namely, *stx*_1a_, *stx*_1c_, *stx*_2a_, *stx*_2b_, *stx*_2c_, and *stx*_2d_ (Table 2).

**TABLE 2:** *In silico* predicted serotype and STEC virulence profile of the 62 STEC genomes.

Isolate ID	Serotype	MLST	STEC virulence barcode
**“Top-7” STEC serotypes**			
CIDM-0310	O157:H7	11	06-00-2A-00-00-00
CIDM-0383	O157:H7	11	06-00-1A-00-00-00
CIDM-0476	O157:H7	11	06-00-1A-2C-00-00
CIDM-0801	O157:H7	11	06-00-1A-2C-00-00
CIDM-0872	O157:H7	11	06-00-1A-2C-00-00
CIDM-1003	O157:H7	11	06-00-1A-2C-00-00
CIDM-1935	O157:H7	7019	06-00-1A-2C-00-00
CIDM-1936	O157:H7	11	06-00-1A-2C-00-00
CIDM-3115	O157:H7	11	06-00-1A-2C-00-00
CIDM-3116	O157:H7	11	06-00-1A-2C-00-00
CIDM-4101	O157:H7	11	06-00-1A-2C-00-00
CIDM-6469	O157:H7	11	06-00-1A-2C-00-00
CIDM-6470	O157:H7	11	06-00-1A-2C-00-00
CIDM-6473	O157:H7	11	06-00-1A-2C-00-00
CIDM-7068	O157:H7	7019	06-00-1A-2C-00-00
CIDM-7108	O157:H7	11	06-00-1A-2C-00-00
CIDM-7116	O157:H7	11	06-00-1A-2C-00-00
CIDM-7541	O157:H7	11	06-00-2A-00-00-00
CIDM-7542	O157:H7	11	06-00-1A-2C-00-00
CIDM-9553	O157:H7	11	06-00-1A-00-00-00
CIDM-9554	O157:H7	11	06-00-1A-2C-00-00
CIDM-9557	O157:H7	11	06-00-1A-00-00-00
CIDM-0384	O26:H11	21	03-00-1A-00-00-00
CIDM-0385	O26:H11	21	03-00-1A-00-00-00
CIDM-1937	O26:H11	29	03-00-2A-00-00-00
CIDM-2890	O26:H11	21	03-00-1A-00-00-00
CIDM-4102	O26:H11	21	03-00-1A-00-00-00
CIDM-4104	O26:H11	21	03-00-1A-00-00-00
CIDM-4106	O26:H11	21	03-00-1A-00-00-00
CIDM-6466	O26:H11	21	03-00-1A-00-00-00
CIDM-7540	O26:H11	21	03-00-1A-00-00-00
CIDM-9556	O26:H11	21	03-00-1A-00-00-00
CIDM-0255	O111:H8	294	07-00-1A-2A-00-00
CIDM-0409	O111:H8	294	07-00-1A-2A-00-00
CIDM-1013	O111:H8	16	07-00-1A-00-00-00
CIDM-6472	O111:H8	294	07-00-1A-2A-00-00
CIDM-9843	O111:H8	294	07-00-1A-2A-00-00
**Non-“Top-7” STEC serotypes**			
CIDM-4105	O128:H2	25	00-00-1C-2B-00-00
CIDM-5426	O128:H2	25	00-00-1C-2B-00-00
CIDM-7100	O128:H2	25	00-00-1C-2B-00-00
CIDM-7539	O128:H2	25	00-00-1C-2B-00-00
CIDM-7543	O128:H2	25	00-00-1C-2B-00-00
CIDM-9842	O128:H2	25	00-00-1C-2B-00-00
CIDM-1995	O91:H14	33	00-00-1A-2B-00-00
CIDM-6468	O91:H14	33	00-00-1A-2B-00-00
CIDM-7994	O91:H14	33	00-00-2B-00-00-00
CIDM-1994	O91:H21	442	00-00-1A-2D-00-00
CIDM-0928	O76:H19	675	00-00-1C-2D-00-00
CIDM-7991	O76:H19	675	00-00-1C-2B-00-00
CIDM-0154	O181:H16	6274	00-T2-1C-2B-00-00
CIDM-4383	O181:H16	6274	00-00-1C-2B-00-00
CIDM-0153	O82:H19	2166	00-02-2A-00-00-00
CIDM-0266	O104:H7	1817	00-00-1C-00-00-00
CIDM-5727	O112:H19	5891	00-00-2A-00-00-00
CIDM-7092	O153:H7	278	00-00-1C-00-00-00
CIDM-1291	O174:H8	13	00-00-1C-00-00-00
CIDM-5427	O2var1:H29^[a]^	515	00-00-2B-2C-00-00
CIDM-9730	O3:H21	297	00-00-1C-00-00-00
CIDM-6471	O37:H10	206	05-00-1A-00-00-00
CIDM-9555	O5:H19	447	00-00-1C-2B-00-00
CIDM-0731	Onovel21:H7^[a]^	2165	00-00-1A-00-00-00
CIDM-2083	Ont:H2	25	00-00-1C-2B-00-00

*[a], O serotypes previously observed in short read data and designated within the EcOH database (Ingle et al., 2016).*

Screening of short-read assembly (NCBI GenBank accession: GCA_000467695.1) and the complete genome (NCBI GenBank accession: CP021339.1) of 95NR1 revealed that multiple isogenic *stx* subtypes could be masked from the *in silico* screen when using draft genomic assemblies as input whereby two additional *stx*_2a_ genes present in the completed genome were missed (Supplementary Table 2). Normalisation of *stx* genes against the *recA* gene demonstrated that majority of the 62 genomes were unlikely to harbour additional isogenic *stx* genes with the exception of two isolates, CIDM-0153 and CIDM-0154. The normalised value for the mean read depth of the implicated *stx* to the mean read depth of *recA* was 2.97 and 2.14 for CIDM-0153 and CIDM-0154, respectively. However, when the + 1 standard deviation value of *recA* was used for normalisation, the value for CIDM-0154 was <2.0 but CIDM-0153 was ≥2.0. Taken together, our results indicated that CIDM-0153 plausibly harboured an additional isogenic *stx*_2a_ operon. On the other hand, CIDM-0154 could harbour an additional *stx*_2b_ but based on described cutoffs, it was equally possible that artefacts resulting from either sequencing or prophage induction during the laboratory cultivation and/or extraction process could have influenced our genome analysis results.

### Analysis of the Most Common Shiga Toxin-Producing *Escherichia coli* Serotypes

Isolates from the “Top-7” STEC serotypes made up more than half of our dataset with the most dominant serotype being O157:H7 (*n* = 22; Figure 1). *In silico* MLST typed 20 STEC O157:H7 isolates as ST11 while two isolates were reported as ST7019. STEC O157:H7 are typically typed as ST11 and a query on EnteroBase (accessed 05/08/2020)^8^ revealed that O157:H7 of ST7019 has also been sighted (*n* = 35) with majority of the isolates originating from Australia (*n* = 32). Three different virulence barcodes (Table 2) were associated with O157:H7 with the majority being 06-00-1A-2C-00-00 (*n* = 17) followed by 06-00-1A-00-00-00 (*n* = 3) and 06-00-2A-00-00-00 (*n* = 2). All O157:H7 isolates harboured a γ1 subtype *eae* consistent with previous reports (Oswald et al., 2000; Blanco et al., 2005) and majority harboured *stx*_1a_ either alone or in combination with *stx*_2c,_ an *stx* genotype common in Australian STEC O157 (Fegan and Desmarchelier, 2002; Mellor et al., 2012, 2013, 2015; Ingle et al., 2019; Pintara et al., 2020).

All STEC O157:H7 genomes were further interrogated with additional serotype-specific typing schemes. Majority (*n* = 20) of O157:H7 isolates possessed the cytosine insertion in *flgF*, suggesting that majority of the isolates were non-motile (Supplementary Table 4). *In silico* LSPA-6 revealed that one O157:H7 isolate belonged to lineage I/II, characterised by an allelic profile of 211111 (Supplementary Table 4), an LSPA-6 lineage typical of STEC O157 from Australia (Mellor et al., 2012, 2013). The other 21 isolates were assigned an allelic profile of 213111 due to the third amplicon targetting *yhcG* yielding an amplicon (392 bases) two bases shorter than a Lineage I designation (394 bases). Interrogation of the *in silico* amplicon sequence revealed that the two base deletion was located at position 91 (A) and 92 (T) of a lineage I *yhcG* amplicon. SNP typing according to the Manning scheme using a reduced discriminatory panel (Yokoyama et al., 2012) indicated that 21 isolates belonged to clade 7 while one isolate belonged to clade 8 with the latter being one of the O157:H7 isolate with a 06-00-2A-00-00-00 barcode designation (Supplementary Tables 2, 4).

Of the O26:H11 isolates (*n* = 10) in our dataset (Table 2), nine represented ST21 while one was typed as ST29. Two virulence barcodes were associated with O26:H11, namely, 03-00-1A-00-00-00 (*n* = 9) and 03-00-2A-00-00-00 (*n* = 1) for ST21 and ST29, respectively. Similar to O26:H11, the STEC O111:H8 isolates (*n* = 5) were split between two sequence types, namely, ST294 (*n* = 4) and ST16 (*n* = 1). This also resulted in two different STEC barcodes, with the ST294 isolates possessing a STEC virulence barcode of 07-00-2A-00-00-00 while the ST16 isolate possessed a barcode of 07-00-1A-00-00-00. Subtypes of *eae* for all O26:H11 (subtype: β1) and O111:H8 (subtype: γ2/θ) isolates were in agreement with previous reports (Oswald et al., 2000; Blanco et al., 2005).

### Uncommon Shiga Toxin-Producing *Escherichia coli* Serotypes Are Less Likely to Encode *eae*

The remaining 25 isolates were spread over 16 serotypes, 15 MLST sequence types, and 12 different assigned barcodes with no overlap of previously assigned barcodes to the “Top-7” serotypes (Table 2). Some non-“Top-7” STEC serotypes in our dataset harboured *stx* subtypes that were detected in their “Top-7” counterparts, with *stx*_1a_ detected in O37:H10 (*n* = 1), O91:H14 (*n* = 2; three isolates in total but one did not harbour *stx*_1a_), O91:H21 (*n* = 1), and Onovel21:H7 (*n* = 1); *stx*_2a_ detected in O112:H19 (*n* = 1) and O82:H19 (*n* = 1); and *stx*_2c_ detected in O2var1:H29 (*n* = 1). Both Onovel21 and O2var1 were novel O serotypes previously observed in short read data and designated within the EcOH database (Ingle et al., 2016). Amongst these eight isolates, four harboured an additional *stx* subtype not encoded by any of the “Top-7” STEC serotypes with *stx*_2b_ harboured by isolates of serotype O2var1:H29 and O91:H14 (*n* = 3) and *stx*_2d_ harboured by an O91:H21 isolate. The remaining 16 isolates belonging to serotypes O128:H2 (*n* = 6), O181:H16 (*n* = 2), O76:H19 (*n* = 2), Ont:H2 (*n* = 1), O104:H7 (*n* = 1), O153:H7 (*n* = 1), O174:H8 (*n* = 1), O3:H21 (*n* = 1), and O5:H19 (*n* = 1) each harboured *stx*_1c_ either alone (*n* = 4) or in combination with *stx*_2b_ (*n* = 11) or *stx*_2d_ (*n* = 1). Only one isolate from a non-“Top-7” STEC serotype harboured *eae* (Table 2).

### Progression to Haemolytic Uraemic Syndrome Predominantly Linked to *stx*_2_ Subtypes

**FIGURE 2:**
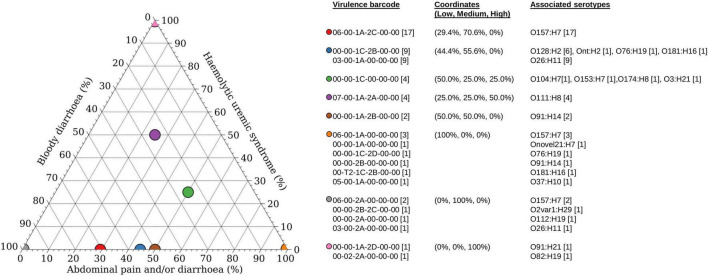
Association of disease outcomes with virulence barcode. Ternary plot of 60 STEC with accompanying clinical information grouped according to virulence barcode and plotted as a percentage against increasing clinical manifestations ranging from low (abdominal pain and/or diarrhoea), medium (bloody diarrhoea), and high (haemolytic uraemic syndrome). Coordinates of each group are listed within parenthesis in the format of (percentage low, percentage medium, and percentage high). Graph was generated using Veusz version 3.2.1 (https://github.com/veusz/veusz).

Of the 60 STEC isolates with accompanying data on clinical presentation, 55 patients presented with either bloody diarrhoea (*n* = 30) or mild clinical manifestations (diarrhoea and/or abdominal pain; *n* = 25). Majority of the patients with mild disease harboured *stx* subtypes that were not typically associated with the progression to HUS (Figure 2), with the exception of a STEC O111:H8 isolate (barcode: 07-00-1A-2A-00-00) and a STEC O76:H19 isolate (barcode: 00-00-1C-2D-00-00). Majority of the *stx*_2a_-positive STECs were isolated from patients with bloody diarrhoea, and none were isolated from patients with mild disease (Figure 2). Five STECs were isolated from patients that progressed to HUS and majority (*n* = 4) possessed an *stx*_2_ subtype (*stx*_2a_ or *stx*_2d_) while the remaining isolate (serotype O104:H7) possessed *stx*_1c_ only. A STEC (serotype O78:H-) harbouring *stx*_1c_ was previously reported to cause HUS in a toddler while other family members remained asymptomatic (Lienemann et al., 2012). Apart from *stx* subtypes, young age was also a risk factor for HUS (Tarr et al., 2005) and could have been a contributing factor to the development of HUS in the report by Lienemann et al. (2012). Two of the four STECs belonged to serotype O111:H8 while the remaining two belonged to serotype O91:H21 and O82:H19, respectively. Interestingly, the O82:H19 isolate was determined to plausibly harbour two copies of the *stx*_2a_ operon (barcode: 00-02-2A-00-00), which could have been a contributory factor to severe disease manifestation as previously observed, albeit in a different STEC serotype (Henning et al., 1998; McAllister et al., 2016).

### Genomic Comparison Ruled Out Community Clusters of Shiga Toxin-Producing *Escherichia coli*

**FIGURE 3:**
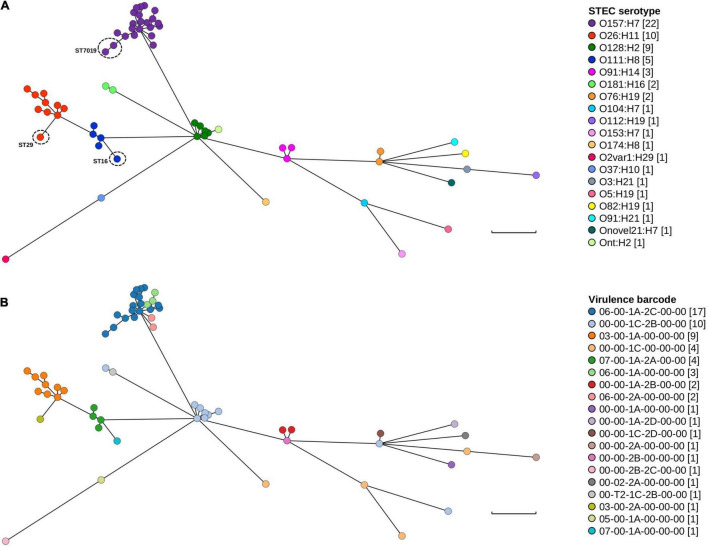
Genomic and STEC virulence diversity of STEC isolates in this study. **(A)** Minimum spanning tree of the 62 STEC genomes generated from wgMLST. **(B)** Minimum spanning tree (wgMLST) with STEC virulence barcode overlaid. Each isolate is represented on the minimal spanning tree as nodes, colour coded according either to serotype or STEC virulence barcode as per the figure key. Branch length between nodes denote number of allelic differences and scale bar represents 900 differences. Tree was generated and visualised on GrapeTree (Zhou et al., 2018).

Only minor genomic diversity within and amongst serotypes based on both MLST and the presence/absence of key STEC virulence factors was documented. Further genomic diversity was interrogated using wg/cgMLST. The difference in the number of loci between the wgMLST and cgMLST schema was 265 fewer loci in the latter scheme. In both wgMLST and cgMLST, each of the 62 isolates possessed a distinct genotype and all isolates belonging to the same serotype clustered together (Figure 3A). There were minor differences in the topology of the minimum spanning tree (MST) between wgMLST (Figure 3) and cgMLST (Supplementary Figure 1) but the general trend of serotype clustering remained the same. When the virulence barcode was overlaid over the wgMLST MST (Figure 3B), it provided a visual snapshot that echoed the aforementioned observation of STECs belonging to the “Top-7” serotypes being more likely to harbour *eae* than non-“Top-7” STEC serotypes and that both *stx*_1c_ and *stx*_2b_ were more likely to be harboured by non-“Top-7” STEC than the “Top-7” serotypes (Table 2 and Figure 3B).

Clustering according to serotype was expected as *wzx* and *wzy* genes were loci for both wgMLST and cgMLST. One STEC genome could not be typed *in silico* (Table 2) but wg/cgMLST grouped it amongst O128:H2, a serogroup that possessed the same MLST sequence type as this particular untypeable STEC. Pairwise comparison (BLASTN) between the draft assembly of this isolate and both *wzx*_O128_ and *wzy*_O128_ revealed that both genes were not assembled. Furthermore, read mapping revealed a mean read depth of 5.61× and 7.56× over *wzx*_O128_ (94.4% coverage) and *wzy*_O128_ (97.8% coverage), respectively. For continuity, this particular isolate was still referred to as Ont:H2. For serotypes that contained multiple MLST sequence types, these isolates could also be easily observed from both wgMLST and cgMLST MST, evident from their longer branch length within their respective serotype cluster (Figure 3 and Supplementary Figure 1). The number of allelic differences within serotypes ranged from 10 to 429 and 1 to 364 while allelic difference between serotypes ranged from 854 to 2,341 and 743 to 2,129 for wgMLST and cgMLST, respectively. The shortest branch length for wgMLST and cgMLST was between two O157:H7 isolates and two O128:H2 isolates, respectively. When typed by wgMLST, the two O128:H2 isolates possessed 12 allelic differences between them while the two O157:H7 isolates possessed six allelic differences when typed by cgMLST. Further interrogation of metadata revealed that both O128:H2 isolates were cultured from patients in the same household in close succession (<1 month) while the two O157:H7 isolates were recovered from unrelated cases 13 months apart in different geographical locations (>200 km).

Core SNP analysis was also performed on isolates belonging to both the “Top-7” serotypes and STEC O128:H2 to supplement wgMLST and cgMLST data. The wide disparity of allelic differences between different serotypes suggested that the utilisation of a single reference genome for all STEC, at least in our dataset, could be problematic and a different, appropriate reference genome was used for each serotype. SNP distance matrices (Supplementary Tables 5–8) showed that the aforementioned two STEC O128:H2 genomes were over 10 SNPs apart while the two STEC O157:H7 isolates were 17 SNPs apart. Taken together, our results suggested that closely related strains could either be inferred from wgMLST followed by core SNP analysis confirmation or inferred directly from cgMLST at the cost of shrinking the number of loci for future analysis.

### Locus of Enterocyte Effacement Sequences and Prophage Associated Non-LEE Effectors Are Present in *eae* Carrying Genomes

**FIGURE 4:**
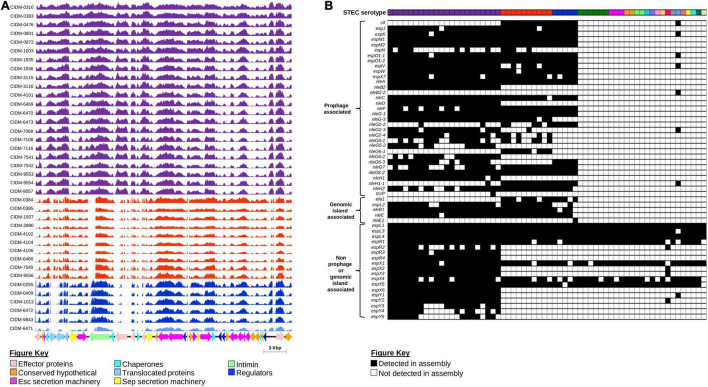
Presence of the LEE Pathogenicity Island and associated effectors amongst the 62 STEC isolates. **(A)** Coverage histogram of LEE-positive isolates across the core LEE sequence of STEC O157 strain Sakai (BA0000007.3) with nucleotide positions on the *x*-axis and coverage per base on the *y*-axis. Histogram bars are colour coded based on serotype listed as per Figures 1, 3. Corresponding coding sequences of the core LEE are colour coded according to function as listed on the Figure key. Figure was generated using Easyfig (Sullivan et al., 2011). **(B)** Presence and absence of non-LEE encoded effectors. All 62 isolates are colour coded according to serotype as per Figures 1, 3. Presence or absence of genes that encode non-LEE effectors are colour coded black and white, respectively.

As *eae* was typically used as a proxy marker for the LEE (Paton and Paton, 1998), presence of the entire core LEE sequence was subsequently confirmed by mapping. Trimmed reads from 38 *eae*-positive STEC were mapped onto the genome of STEC O157 strain Sakai (BA000007.3; positions: 4,589,382–4,623,687). All *eae*-positive genomes had reads covering majority of the LEE albeit at variable read depths (Figure 4A). Coverage over the entire core LEE of Sakai ranged from 99.8 to 100%, 86.41 to 87.11%, 84.26 to 85.19%, and 81.60% for serotypes O157:H7, O26:H11, O111:H8, and O37:H10, respectively. Genes that were required to build the T3SS apparatus all had read coverage confirming that the T3SS apparatus of these 38 isolates were intact. The main regions of non-coverage were over the 3′ end of *eae*, which was discriminatory for subtypes (Blanco et al., 2005) and over genes encoding effector proteins (Figure 4A).

While the LEE pathogenicity island harboured genes that encoded effector proteins, there were other effector proteins not encoded within the LEE but were secreted by this T3SS (Tobe et al., 2006). All LEE-positive “Top-7” STECs harboured significantly more non-LEE effector genes than both the sole LEE-positive non-“Top-7” STEC and LEE-negative isolates (Figure 4B and Supplementary Table 9). Some non-LEE effector genes were also detected in LEE-negative STEC, but these were effector genes not previously associated with either prophages or known genomic islands (Figure 4B). On the other hand, only LEE-positive STEC possessed a suite of non-LEE effector genes that were prophage associated (Figure 4B).

### Scanning for Additional Virulence Factors Found No Hybrid Pathotypes Apart From *eae*-Positive Shiga Toxin-Producing *Escherichia coli*

**FIGURE 5:**
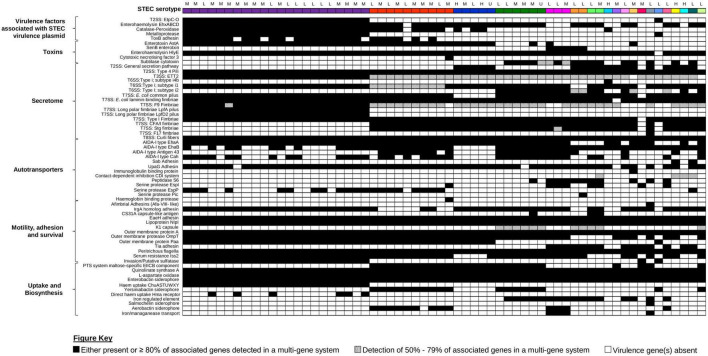
Presence/absence of other virulence factors amongst the 62 STEC isolates. All 62 isolates, represented on the first row, are colour coded according to serotype as per Figures 1, 3. Presence or absence of virulence factor-associated genes are colour coded black and white, respectively. Virulence factors that consisted of multiple genes were considered present when ≥80% of the associated genes listed in the ecoli_vf database are present. If only 50–79% of the associated genes were present, the virulence factor was colour coded with black dots. If less than 50% of associated genes were present, the virulence factor was inferred as absent and colour coded in white. Associated clinical manifestations of abdominal pain and/or diarrhoea (L), bloody diarrhoea (M), and haemolytic uraemic syndrome (H) for each isolate are listed above serotype. Isolates with no appended data of clinical manifestation data are listed as (U).

Presence of additional virulence factors was inferred from the assemblies of the 62 STEC against the Ecoli_VF Database^9^ (Figure 5 and Supplementary Table 9). Apart from genes encoded by the LEE pathogenicity island harboured by EPEC, none of our STEC harboured genes were associated with signature virulence factors of other *E. coli* pathotypes. Interestingly, the additional virulence genes detected were stratified according to serotype (Figure 5). Similar to *eae*, *ehxA* was used as a proxy marker for the presence of a pO157-like virulence plasmid in STEC (Paton and Paton, 1998). Amongst the 62 STEC, *ehxABCD* was detected in the genomes of 52 isolates, indicative that majority of our STEC likely harboured a STEC virulence plasmid (Figure 5). Virulence genes associated with the STEC virulence plasmid were also detected, but majority of these virulence genes were present in isolates belonging to serotype O157:H7.

Apart from the production of Stx, other toxin genes (*hlyE*, *astA*, *cnf3*, and *senB*) that could either contribute to the manifestation of diarrhoea (Yatsuyanagi et al., 2003) or invasion (Knust and Schmidt, 2010) were also detected in the genomes of the STEC in our dataset (Figure 5). Majority of the non-“Top-7” serotypes (*n* = 17) also contained the genes (*subAB*) that encode the subtilase cytotoxin (Paton et al., 2004). This holotoxin is typically associated with STEC that do not harbour the LEE pathogenicity island and binds to a sialic acid that is present in most mammals but not synthesised by humans (Paton et al., 2004; Byres et al., 2008). However, this sialic acid could be incorporated by humans *via* diet, and it has been suggested that dietary choices could contribute to disease in STEC producing this toxin (Byres et al., 2008).

A suite of virulence genes that contributed to colonisation were also detected in the genomes in the 62 STEC. These included genes associated with the secretome, as well as outer membrane proteins that mediated adhesion to host cells, invasion, and binding to host produced extracellular matrix (Figure 5 and Supplementary Table 8). While the genes associated with the Type I fimbriae was detected in all STEC O157:H7 isolates, interrogation of the switch upstream of *fimA* revealed that all STEC O157:H7 isolates possessed the 16-bp deletion consistent with published observations that the Type I fimbriae was not expressed in this serotype (Shaikh et al., 2007). While genes associated with the ETT2 T3SS was detected in majority of our STEC, this secretion system has been reported to be defective due to mutational attrition (Ren et al., 2004), and it was likely to be defective in our STECs as well. Apart from colonisation, a pathogen would also need to be able to scavenge for iron as restriction of iron availability is a mechanism of the innate immunity. In addition, iron is also a negative regulator for Stx1a production (Calderwood and Mekalanos, 1987) and the ability to effectively scavenge iron could have implications on toxin production amongst Stx1a-positive STEC. All STEC isolates possessed the genes involved in the biosynthesis of the Enterobactin siderophore (Figure 5 and Supplementary Table 8) while some also harboured the genes for stealth siderophore synthesis (Fischbach et al., 2006), namely, aerobactin (*n* = 13, 20.97%), salmochelin (*n* = 1, 1.61%), and yersiniabactin (*n* = 18, 29.03%). Apart from siderophores, some of our STECs also harboured genes for other iron uptake mechanisms ranging from a receptor for haem acquisition to systems for direct haem uptake (Figure 5).

## Discussion

This study examined the added value of WGS in improving the resolution of public health laboratory surveillance of STEC in a low-incidence country. It has been previously shown that WGS can be used to great effect in tracking outbreaks of STEC O157 (Dallman et al., 2015; Jenkins et al., 2019). As there were no large outbreaks of STEC during our study period, we were unable to provide additional commentary on the usefulness of WGS in outbreak tracking. Routine public health surveillance has been focussed on utilising SNP-based analysis for the transmission tracking of pathogens of interest. As STEC are a group of pathogens spanning multiple serotypes, MLST types, and possibly being geographically and genetically distinct, the utility of a potentially distant reference genome *via* SNP analysis could perturb the resolution afforded by SNP analysis. To address this, we employed and adapted a publicly available wgMLST/cgMLST scheme to infer the genomic diversity of STEC in our dataset. The utility of cgMLST has been previously shown to be a scalable and a viable alternative approach for high-resolution analysis (Pearce et al., 2018) and has been utilised to infer genetic relatedness of STEC of interest to public health (Ferdous et al., 2016; Lang et al., 2019; Smith et al., 2019). Despite our small dataset, this approach was able to corroborate with metadata on isolates that have an epidemiological link, albeit clearer at the cgMLST level rather than the wgMLST level as the latter could also capture isolates that were unlikely to be linked but still in circulation in the environment. Regardless, wgMLST/cgMLST may inform further downstream core SNP analysis, which is typically used in public health surveillance and outbreak tracking.

Shiga toxin-producing *Escherichia coli* O157:H7 dominated our dataset, a result concordant with previous epidemiological studies performed in Australia (Vally et al., 2012; Ingle et al., 2019). Our STEC O157:H7 predominantly carried *stx*_1a_ only, *stx*_2c_ only, or both *stx*_1a_ and *stx*_2c_ in combination, similar to previous reports (Fegan and Desmarchelier, 2002; Mellor et al., 2012, 2013, 2015). Interestingly, majority of the STEC O157:H7 isolates showed an allelic pattern of 213111 instead of the 211111 typical of Australian O157:H7 when typed by LSPA-6 (Mellor et al., 2013), a difference attributed to a 2-bp deletion in *yhcG*. We inferred hypervirulence for STEC O157:H7 using the Manning SNP typing scheme whereby hypervirulent isolates belonged to clade 8 (Manning et al., 2008). Similar to previous reports (Mellor et al., 2012), majority of the STEC O157:H7 in our study were typed as clade 7 of the Manning scheme while a single isolate was typed as a hypervirulent clade 8 strain. Hypervirulent STEC O26:H11 strains belonging to ST29 have been shown to harbour the *stx*_2a_ operon (Bielaszewska et al., 2013). The ST29 O26:H11 in our dataset also harboured the *stx*_2a_ gene. While MLST is generally embedded in routine genomic analysis in prokaryotic pipelines, the Manning SNP clade is not, and valuable information on potential hypervirulent STEC O157:H7 could be lost.

Whole genome sequencing has improved the resolution of subtyping of both *stx*_1_ and *stx*_2_, which supported additional risk assessment as certain *stx* subtypes, particularly *stx*_2_, are more likely to lead to HUS (Friedrich et al., 2002; Orth et al., 2007). Indeed, our STEC isolated from patients that progressed to HUS predominantly harboured either *stx*_2a_ or *stx*_2d_ subtypes, which had been previously associated with a higher likelihood for progression to HUS (Friedrich et al., 2002; Orth et al., 2007). The most common *stx* subtype detected amongst our STEC dataset was *stx*_1a_, which was detected in both “Top-7” and non-“Top-7” serotypes. In addition, subtypes *stx*_1c_ and *stx*_2b_ were more associated with the non-“Top-7” STEC in our dataset. This led to the development and evaluation of a STEC virulence barcode, which, when used in tandem with wgMLST/cgMLST visualisation, provided a visual snapshot of pertinent virulence factors relevant for routine surveillance and tracking the *stx* genotypes. In addition, this barcode also helps to infer multiple isogenic *stx* in a genome from short read sequencing data. The encoding of multiple isogenic *stx* has been implicated with disease severity such as in the latter phase of the 1996 STEC O111:H- outbreak in South Australia, when the isolates appeared to be more virulent (Henning et al., 1998). This increase in virulence was subsequently attributed to the carriage of additional *stx*_2a_ operons, which was confirmed *via* sequencing (McAllister et al., 2016; Forde et al., 2019). Similarly, one STEC isolated from a HUS patient in our dataset also plausibly harboured an additional *stx*_2a_ operon as determined by short read sequencing. However, the limits of utilising short read sequencing meant that we were unable to definitively confirm the multiple carriage of *stx*_2a_ prophages, with confirmation only possible with the utilisation of long read sequencing (Forde et al., 2019). In addition, we are also unable to definitively comment if this was the sole reason that led to HUS. Nonetheless, this aforementioned case, along with 1996 outbreak, provided the impetus that while tracking *stx* subtypes is important, tracking or inference of multiple copies of the same *stx* subtypes, especially of the more virulent subtype, could be equally valuable.

While the STEC virulence barcode could be useful for surveillance and risk assessment of virulence, it is unlikely to function as a molecular predictor of disease outcome. Additional factors that could also contribute to the overall pathogenicity for a STEC infection should be acknowledged. Host risk factors, like age and immune system status, have been previously associated with adverse outcomes (Tarr et al., 2005). In addition, an individual’s gut microbiota could also serve as a genetic pool for new STEC as it has been previously shown in germ-free mice models that co-colonisation with STEC and commensal *E. coli* led to a synergistic increase in disease state (Goswami et al., 2015), a testament to horizontal transfer of its namesake virulence factor.

With particular *stx* subtypes (*stx*_2a_ and *stx*_2d_) more likely to progress to HUS present in the minority of our dataset, our study corroborated with previous reports on STEC, albeit with a STEC O157 bias, that reduced virulence could be due to both *stx* genotype and *E. coli* genetic background (Mellor et al., 2015). The predominant subtype detected in our dataset was Stx1a, which is known to be less cytotoxic than the subtypes Stx2a, Stx2d, and Stx2c (Friedrich et al., 2002; Orth et al., 2007). Previous reports, albeit with a STEC O157:H7 bias, have also shown that Stx1a was the predominant Stx subtype circulating in Australia (Mellor et al., 2012, 2015; Ingle et al., 2019; Pintara et al., 2020). While the lower potency of Stx1a could explain the lower virulence of STEC in Australia, we would like to posit a possible genomic contributing factor to reduced virulence, at least in Stx1a-producing STEC. Expression of Stx1a has been previously shown to be also under the influence of iron, where iron limitation leads to increased expression of the toxin (Calderwood and Mekalanos, 1987). Our STEC all possessed genes encoding the synthesis of the enterobactin siderophore, an iron scavenging molecule to obtain iron from its environment. As a countermeasure, lipocalin-2 is produced by the human host, which binds iron-bound enterobactin, preventing iron uptake by bacteria (Goetz et al., 2002). Majority of our STEC isolates, however, also encoded genes that could synthesise other stealth siderophores, which would facilitate the evasion of lipocalin-2-mediated iron starvation (Fischbach et al., 2006). However, an antimicrobial peptide produced by humans, LL-37, has been shown to be able target stealth siderophores (Zsila and Beke-Somfai, 2020). A counter to LL-37 activity lies in the production of the outer membrane protease OmpT, which can degrade LL-37 (Thomassin et al., 2012), of which its associated gene was detected in majority of our STEC. The detection of genes encoding toxins capable of releasing haem, along with systems for the direct uptake of haem in our STECs, also provided an additional layer for iron sequestering required for biological activity. Indeed, the interplay of various virulence factors indicated that iron starvation could be evaded, perturbing the production of Stx1a *via* the iron regulation pathway in the process. In a geographical location whereby the most clinically dominant serotype typically harbours the genes encoding Stx1a, this possible negation of toxin production, along with the lower potency of Stx1a, could explain why the incidence of STEC related illness in Australia is lower than our international counterparts.

To our knowledge, this is the first report of prophage-associated non-LEE effectors in STEC serotype O37:H10. Isolates belonging to serotype O37:H10 has been previously observed to harbour the LEE encoded *eae* gene but were not further subtyped (Brett et al., 2003). Our results showed that similar to the *eae*-positive “Top-7” serotypes, this particular isolate also harboured non-LEE effector genes that were associated with prophages. These prophage-associated effector genes are typically located in a conserved location, downstream of genes encoding the phage tail fibres (Tobe et al., 2006), and could be transferred to other susceptible *E. coli* (Creuzburg et al., 2005). The presence of these genes in this non-prolific STEC serotype furthered the support that the (pro)phage metagenome is indeed linked to the pathogenesis of bacteria harbouring an injectisome Type 3 secretion system (Tobe et al., 2006).

It is important to note that our dataset only represented 33.33% of STEC notifications across NSW. This limitation reflects challenges of the recovery of STEC isolates from stool samples further complicated by the horizontal acquisition and potential loss of its namesake virulence factor in an *E. coli* genomic background (Senthakumaran et al., 2018). In addition, our current laboratory standard operating procedure limits the number of colonies selected for further *stx* detection to three. This approach could also contribute to relatively low recovery of STEC and an increase in the number of isolates picked could have a positive effect on STEC recovery (van Duynhoven et al., 2008). Nonetheless, a systemic update focussing on improvements to STEC culturing could provide additional circumstantial evidence to strengthen our postulation of genomic factors working in concert, leading to reduced virulence.

## Conclusion

The genomic investigation of STEC over a period of 30 months in New South Wales, Australia highlighted the added value of high-resolution laboratory surveillance in a low-incidence setting. It offered detailed characterisation of the predominant harbouring of *stx* genes and hypervirulent clades/sequence types, which formed the minority of STEC isolates. The genetic background of the iron uptake systems, along with interactions with other virulence factors, could be a contributing factor in limiting the virulence in Stx1a-producing STEC, which made up the majority of the STEC in our study. Our findings emphasise the importance of sustained culture-based genomic surveillance for this group of genomically malleable pathogens of public health interest.

## Data Availability Statement

The datasets presented in this study can be found in online repositories. The names of the repository/repositories and accession number(s) can be found in the article/Supplementary Material.

## Author Contributions

VS, AA, PH, RK, and ES: study concept and design. ES, RK, MG, and AA: data analysis. ES, MG, RK, AA, MV, BH, and VS: manuscript writing, editing, and/or reviewing. All authors read and approved the manuscript.

## Conflict of Interest

The authors declare that the research was conducted in the absence of any commercial or financial relationships that could be construed as a potential conflict of interest.

## Publisher’s Note

All claims expressed in this article are solely those of the authors and do not necessarily represent those of their affiliated organizations, or those of the publisher, the editors and the reviewers. Any product that may be evaluated in this article, or claim that may be made by its manufacturer, is not guaranteed or endorsed by the publisher.

## Footnotes

1
https://github.com/tseemann/abricate

2
https://github.com/phac-nml/ecoli_vf

3
https://github.com/tseemann/mlst

4
https://www.cgmlst.org/ncs/schema/5064703/

5
https://github.com/tseemann/snippy

6
https://github.com/tseemann/snp-dists

7
https://users.soe.ucsc.edu/~kent/

8
https://enterobase.warwick.ac.uk

9
https://github.com/phac-nml/ecoli_vf

## Supplementary Material

Supplementary Figure 1 Minimum spanning tree of the of the 62 STEC genomes generated from cgMLST. Each isolate is represented on the minimal spanning tree as nodes, colour coded according either to serotype or STEC virulence barcode as per the figure key. Branch length between nodes denote number of allelic differences with scale bar represent 1,000 differences. Tree was generated and visualised on GrapeTree (Zhou et al., 2018).
