# Supplementary figures and images for: Added Value of Genomic Surveillance of Virulence Factors in Shiga Toxin-Producing Escherichia coli in New South Wales, Australia
Source: Front Microbiol. 2021 Dec 23;12:713724. doi: 10.3389/fmicb.2021.713724 (PMC8733641; doi:10.3389/fmicb.2021.713724)

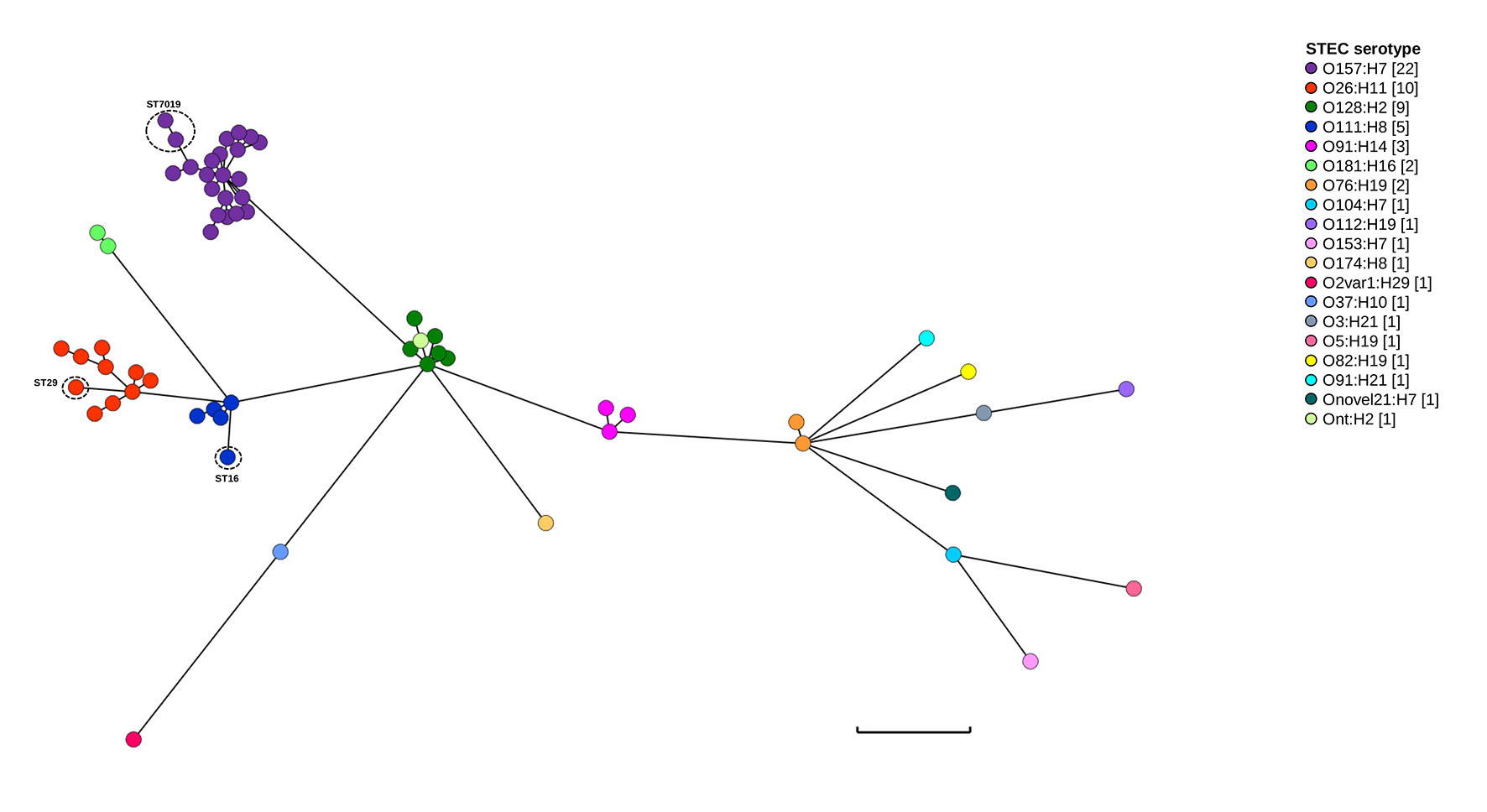

Supplement: Supplementary Figure 1 — Minimum spanning tree of the of the 62 STEC genomes generated from cgMLST. Each isolate is represented on the minimal spanning tree as nodes, colour coded according either to serotype or STEC virulence barcode as per the figure key. Branch length between nodes denote number of allelic differences with scale bar represent 1,000 differences. Tree was generated and visualised on GrapeTree (Zhou et al., 2018). [file Image_1.JPEG]
